# Evaluation of a CTA-based convolutional neural network for infarct volume prediction in anterior cerebral circulation ischaemic stroke

**DOI:** 10.1186/s41747-021-00225-1

**Published:** 2021-06-24

**Authors:** Lasse Hokkinen, Teemu Mäkelä, Sauli Savolainen, Marko Kangasniemi

**Affiliations:** 1 HUS Medical Imaging Centre, Radiology, University of Helsinki and Helsinki University Hospital, P.O. Box 340 (Haartmaninkatu 4), FI-00290 Helsinki, Finland; 2 Department of Physics, University of Helsinki, P.O. Box 64, FI-00014 Helsinki, Finland

**Keywords:** Computed tomography angiography, Stroke, Deep learning, Machine learning, Convolutional neural network

## Abstract

**Background:**

Computed tomography angiography (CTA) imaging is needed in current guideline-based stroke diagnosis, and infarct core size is one factor in guiding treatment decisions. We studied the efficacy of a convolutional neural network (CNN) in final infarct volume prediction from CTA and compared the results to a CT perfusion (CTP)-based commercially available software (RAPID, iSchemaView).

**Methods:**

We retrospectively selected 83 consecutive stroke cases treated with thrombolytic therapy or receiving supportive care that presented to Helsinki University Hospital between January 2018 and July 2019. We compared CNN-derived ischaemic lesion volumes to final infarct volumes that were manually segmented from follow-up CT and to CTP-RAPID ischaemic core volumes.

**Results:**

An overall correlation of *r* = 0.83 was found between CNN outputs and final infarct volumes. The strongest correlation was found in a subgroup of patients that presented more than 9 h of symptom onset (*r* = 0.90). A good correlation was found between the CNN outputs and CTP-RAPID ischaemic core volumes (*r* = 0.89) and the CNN was able to classify patients for thrombolytic therapy or supportive care with a 1.00 sensitivity and 0.94 specificity.

**Conclusions:**

A CTA-based CNN software can provide good infarct core volume estimates as observed in follow-up imaging studies. CNN-derived infarct volumes had a good correlation to CTP-RAPID ischaemic core volumes.

## Key points

- A computed tomography angiography (CTA)-based convolutional neural network (CNN) can predict infarct volume in anterior circulation ischaemic stroke.
- A CTA-based CNN estimates of ischaemic lesion volumes correlated well with infarct volumes measured from follow-up computed tomography images.
- Our method had a good correlation with computed tomography perfusion-RAPID estimated infarct core volumes.

## Background

Artificial intelligence applications have shown promise in the detection of acute ischaemic stroke (AIS) from computed tomography (CT)-based studies. Published studies to date have mainly focused on the detection of anterior circulation AIS from non-contrast CT scans, large vessel occlusions from non-contrast CT or CT angiography (CTA) and automated Alberta Stroke Programme Early CT Score (ASPECTS) scoring from non-contrast CT scans. Many of these studies have looked at the performance of commercially available stroke diagnosis software. According to a recent review article, the current literature reports a broad sensitivity metrics for the detection of AIS, the mean sensitivity being 68% [1].

Currently, perfusion imaging is being widely used in determining ischaemic core (unsalvageable tissue) and penumbra (salvageable tissue) volumes. A CT perfusion (CTP) study is faster and more widely available than the current gold standard magnetic resonance imaging and provides acceptably accurate estimates to aid in treatment selection. CTP estimations of ischaemic core and penumbra are used in patient selection for thrombolytic therapy and mechanical thrombectomy [2, 3]. Large studies demonstrating the safety and efficacy of thrombolytic therapy in the extended (4.5–9 h) time window have used ischaemic core volume thresholds of 70 and 100 mL to determine eligibility for thrombolytic therapy [4, 5]. Recent studies have evaluated the safety of intravenous thrombolysis in patients with AIS of unwitnessed onset at 4.5–24 h since last known well and also in patients with wake-up stroke in a treatment window that was 9 h after the midpoint of the time they fell asleep to the time they woke with symptoms [6]. The DAWN study demonstrated the efficacy and safety of thrombectomy in patients with occlusion of the intracranial internal carotid artery or proximal middle cerebral artery in patients who had last been known to be well 6–24 h earlier and who had a mismatch between the severity of the clinical deficit and the infarct core [3]. This means that patients were treated up to almost 16 h after the time the patient was last known to be well. However, availability issues in smaller hospitals, increased radiation dose and susceptibility to motion artefacts are downsides of CTP. CTA on the other hand is more readily available and provides the possible presence and site of arterial occlusion.

Three studies have investigated the use of artificial intelligence in detecting anterior circulation AIS from single-phase CTA source images (CTA-SI) with promising results. Our previous study showed that an acute ischaemic stroke can be detected with 3D convolutional neural network-based software from CTA-SI with high sensitivity and specificity [7]. A study by Sheth et al. showed that brain symmetry can be leveraged to accurately detect ischaemic related changes and large vessel occlusions from CTA-SI [8]. Hilbert et al. demonstrated that functional and reperfusion outcome of treatment could be predicted from a CTA-SI volume reduced to a single axial plane using maximum intensity projection [9]. None of these earlier publications have compared how these changes would correlate to infarct size and location from follow-up imaging studies.

In this study, our aim was to determine the accuracy of our previously developed convolutional neural network (CNN) model in final infarct volume prediction from CTA-SI in anterior circulation ischaemic stroke in patients treated conservatively or with intravenous thrombolysis. We also sought to determine the CNNs anatomical accuracy using ASPECTS regions and to compare the CNNs performance to a widely used, commercially available software (CTP-RAPID, iSchemaView, Menlo Park, CA) in infarct core estimation in the acute setting to evaluate the efficacy of our method in treatment selection. This could be useful especially in hospitals where CTP may not be available, or, as CTA is readily acquired in current AIS imaging protocols, the CNN prediction could complement a study where CTP reading has proven uncertain or inconclusive.

## Methods

Helsinki University Hospital ethical committee approved this retrospective study and patients’ informed consent was waived.

### Study population

We retrospectively studied the clinical and imaging findings of consecutive stroke suspected cases that presented to Helsinki University Hospital between January 2018 and July 2019. Ninety-one patients met the following inclusion criteria: (1) stroke code activated, (2) admission stroke protocol imaging performed using fast CTA-SI acquisition protocol and CTP, (3) patient received either thrombolytic therapy or supportive treatment and (4) a discernible infarct on follow-up non-contrast CT study performed no later than 6 days after the onset of symptoms. Patients treated with mechanical thrombectomy were excluded from this study. Six patients were excluded due to failed perfusion studies and two patients due to poor quality follow-up CT leaving a total of 83 patients for the analysis. The mean age of these patients was 69 years (SD 11.6, range 41–92), 49 were male and 34 were female as shown in Table 1. Thirty-seven patients received thrombolytic therapy.

**Table 1:** Patient characteristics

Number of patients	83
Age (years), mean (SD, range)	68.7 (11.6, 41–92)
Male sex, number (%)	49 (59)
Time from symptom onset to start CT	
< 9 hours	57
> 9 hours	26
Intravenous thrombolysis, number (%)	37 (45)
Infarct lesion volumes (mL), mean (SD, range)	
CNN output	36 (48, 0–241)
CTP-RAPID infarct core (CBF < 30%)	23 (36, 0–170)
Final infarct volume	52 (67, 1–301)

*CT* Computed tomography, *CNN* Convolutional neural network, *CTP* CT perfusion, *CBF* Cerebral blood flow, *SD* Standard deviation

### Image acquisition and pre-processing

All patients were imaged in the acute setting using a Somatom Definition Edge (Siemens Healthineers, Erlangen, Germany) 128-slice CT scanner. The CTA imaging parameters were tube voltage 120 kVp, reference current time 150 mAs, pitch 1.3, reconstruction kernel I30f and slice thickness/increment 0.75/0.5 mm. The iodine concentration of the contrast agent was 350 mg/mL with an amount of 50 mL and injection rate of 5 mL/s. The timing of the scan was 12 s after time to peak of the test bolus. CTP imaging parameters were tube voltage 80 kVp and reference current time 120 mAs, pitch 0.5 and reconstruction kernel H20f with a contrast agent amount of 45 mL and injection rate of 6 mL/s. The follow-up CT studies used to assess final infarct volumes were performed with various CT scanners in different hospitals. The majority of the follow-up studies (73%) were performed 24 h after admission with a median time interval of 36 h (interquartile range [IQR] 12–36 h). Images were anonymised and stored on a server running the Extensible Neuroimaging Archive Toolkit version 1.1.6 [10].

**Fig. 1:**
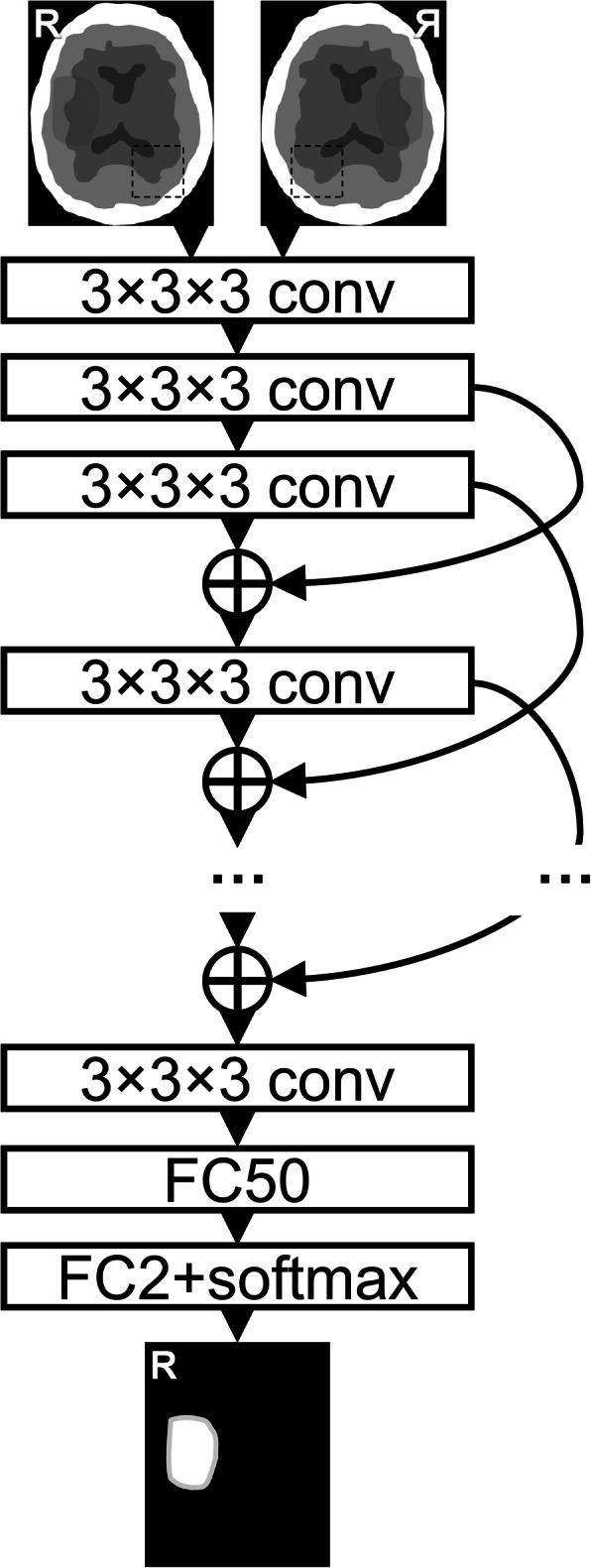
The neural network architecture consisted of 40 three-dimensional convolutional layers (conv) with valid padding and exponential linear unit activation. Skip-connections (curved arrows) with concatenations (+) were used with appropriate cropping. These were followed by two fully connected layers (1 × 1 × 1 convolutions, FC) with 50 and 2 output neurons, and rectified linear unit and softmax activation, respectively. In the end, the predicted patches were stitched together to produce a lesion segmentation of the same size as the input

The infarcted regions were segmented on follow-up CT scans in consensus by a senior neuroradiologist (M.K.) and a radiologist in training (L.H.), with over 20 and 5 years of experience, respectively, using 3D Slicer image processing and visualisation platform [11]. The CNN was trained on CTA-SI volumes of 20 non-stroke and 20 stroke patients with manually delineated lesion targets. It consisted of a two-channel input, a total of 40 3D convolutional layers with 3 × 3 × 3 kernel size, 16 filters each and valid padding, followed by a fully connected layer with 50 neurons and a two-neuron output (lesion/background) with softmax activation producing voxel-by-voxel lesion presence confidences. Skip connections passing single layers were used to encourage gradient propagation. The network was fed with 147 × 147 × 147-voxel sub-volumes with equal number of stroke lesion positive and negative sub-volumes in each batch. The second input channel was the corresponding (left-right-mirrored and registered) sub-volume from the contra-lateral hemisphere. The model was trained using batch-size 8 and Adam optimiser for 30 epochs after which validation loss, calculated on a separate set of ten CTA-SI volumes, stopped improving. The network was implemented using Keras library version 2.2.4 [12] and Tensorflow version 1.12.0 [13]. A graphical representation of the CNN architecture is provided in Fig. 1.

### Study design

The CNNs performance regarding anatomical accuracy against expert segmentation was evaluated from follow-up CT studies by ASPECTS anatomical regions visually, i.e., did the CNN predicted lesions’ locations match the final infarct locations within the ASPECTS regions in the middle cerebral artery territory. ASPECTS is a well-established method for quantitative topographic evaluation of middle cerebral artery stroke. Individual regions were labelled ‘positive’ or ‘negative’ for ischaemic changes by a radiologist (L.H.), as determined by the CNN from acute phase CTA-SI and by manual segmentations from follow-up CT. The manual segmentations were considered as ground truths. Confusion matrices, sensitivity, specificity, and Sørensen–Dice similarity coefficient were then calculated from the regions’ true or false labelling.

The CNNs performance was compared against a commercial software (CTP-RAPID) that derives the infarct core volume from CTP, as this is a validated and widely used method for treatment selection. For this comparison, the effect of two clinically relevant time windows (from symptom onset to start of imaging protocol) on CNN output accuracy was tested.

Only lesions in the affected cerebral hemisphere detected by the CNN were selected for the volumetric analysis with a volume threshold > 0.1 mL and a CNN output (probability) threshold ≥ 0.5 for lesion inclusion. False positive lesions in the contralateral hemisphere or posterior fossa were discarded from the analysis. This approach was chosen because in anterior circulation AIS the affected hemisphere can usually be deducted from clinical presentation. A visual and volumetric analysis of false positive lesions in the contralateral hemisphere and posterior fossa that were discarded from the volumetric analysis was performed with the same lesion inclusion thresholds indicated above.

### Statistical analysis

Linear regression models between the CNN-derived volume outputs, manually segmented final infarct volumes and CTP-RAPID ischaemic core volumes (defined by relative cerebral blood flow (CBF) < 30%) were calculated. Pearson correlation coefficients (*r*) were calculated to evaluate the correlation of CNN- and CTP-RAPID-derived volumes against final infarct volumes and CNN-derived volumes to CTP-RAPID core volumes. Confidence intervals (CI) for the *r* values were calculated using the bootstrap method by repeating resampling with replacement 10^5^ times. Bland-Altman plots of agreement between infarct volume estimates and final infarct volumes and between CNN- and CTP-RAPID-derived estimates were also calculated. The calculations were performed using MATLAB version 2018b (MathWorks, Natick, MA, USA).

## Results

**Fig. 2:**
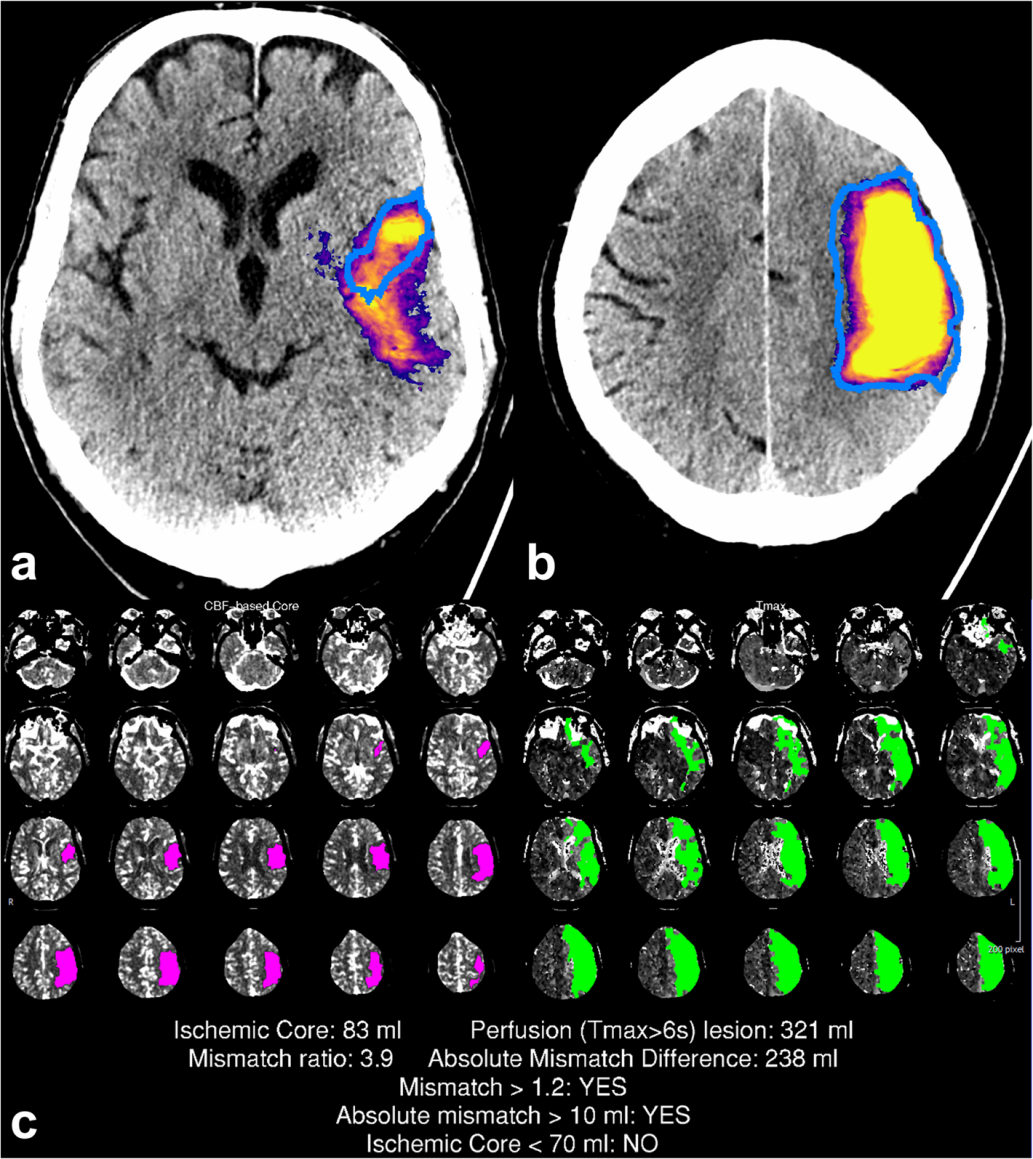
A large infarct correctly detected by the convolutional neural network (CNN). The CNN prediction included the final infarct (blue outline) and a part of the penumbra. Representative slices of the CNN predictions (**a**, **b**) with corresponding computed tomography perfusion RAPID (CTP-RAPID) report for comparison (**c**). The purple-orange-yellow colourmap depicts CNN output probability. Reported volumes: CTP-RAPID ischaemic core 83 mL, CNN 150 mL, and final infarct 150 mL

A representation of the CNN output with manual segmentation and corresponding CTP-RAPID output is shown in Fig. 2.

**Fig. 3:**
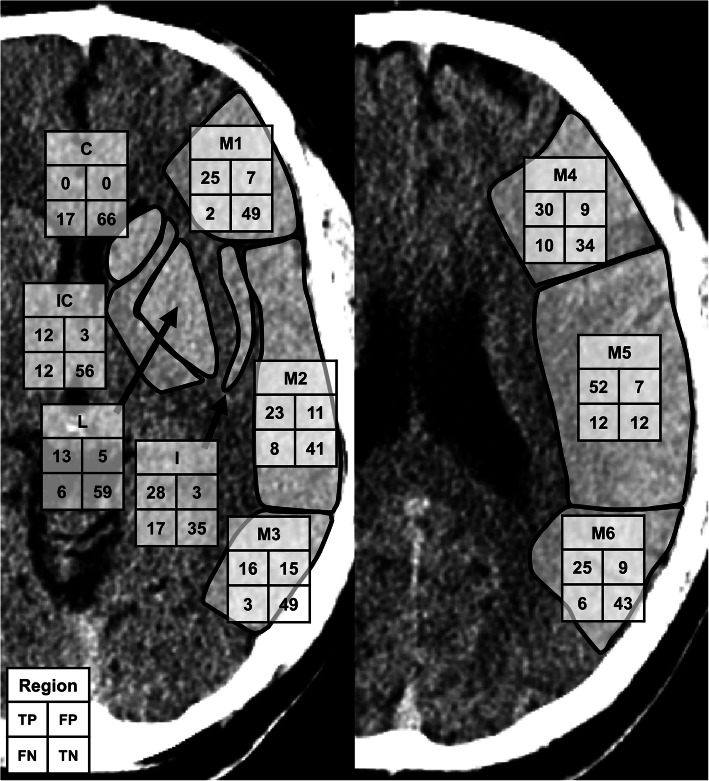
Confusion matrices for the convolutional neural network (CNN) detections by the ten individual Alberta Stroke Programme Early CT Score (ASPECTS) regions (M1–6, C, IC, L, and I) for 83 patients. The total sums from the left and right hemispheres (whichever was the infarcted side) were combined for true positives (TP), true negatives (TN), false positives (FP), and false negatives (FN)

ASPECTS regions were used to evaluate the anatomical accuracy of the CNN against expert segmentations of final infarct volumes. A total of 830 regions were evaluated. The CNN outputs had a sensitivity of 0.71, a specificity of 0.87 and an accuracy of 0.80. The Sørensen–Dice similarity coefficient was 0.73. The patient-wise median (IQR) for accuracy was 0.8 (0.7–0.9), and for the Sørensen–Dice similarity coefficient 0.67 (0.40–0.89). A representation of true negatives, true positives, false negatives and false positives by ASPECTS regions can be found in Fig. 3.

Lesion volumes from CNN outputs and manual segmentations of final infarcts were calculated from all lesions in the affected cerebral hemisphere. CTP-RAPID ischaemic core estimations were reported as calculated by the software. The infarct lesion volumes shown in Table 1 ranged from 0–241 (mean 36, SD 48) mL in the CNN outputs and from 1–301 (mean 52, SD 67) mL in the manual segmentations. The reported CTP-RAPID ischaemic core volumes, as defined by CBF < 30%, ranged from 0–170 (mean 23, SD 36) mL.

**Fig. 4:**
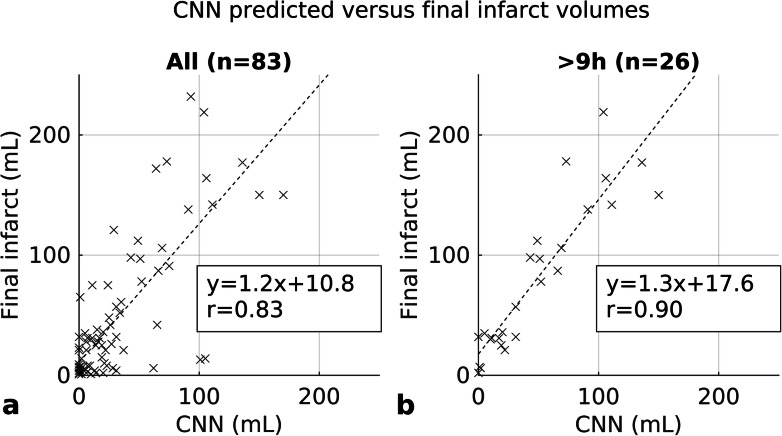
Infarct lesion volume correlation between convolutional neural network (CNN) predictions based on acute phase computed tomography angiography (CTA) and final infarct volumes. All patients (**a**). Patients imaged more than 9 h after symptom onset (**b**)

**Fig. 5:**
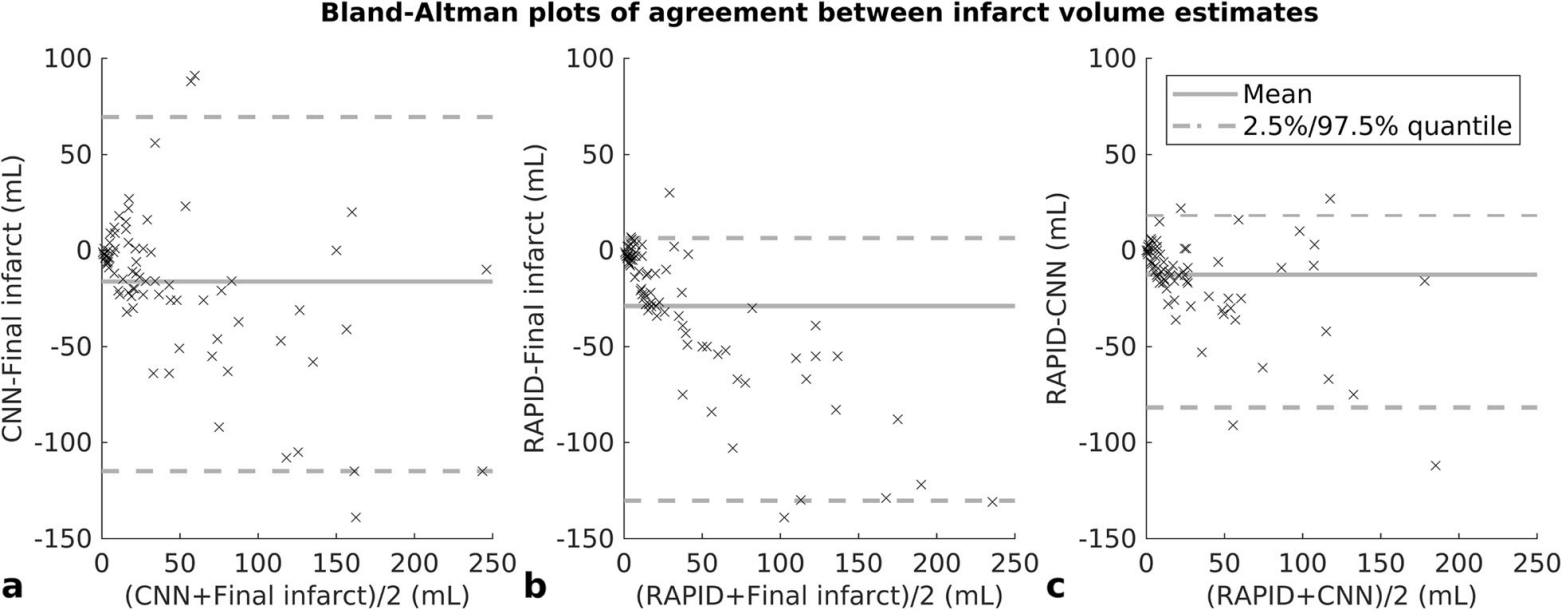
Bland-Altman plots of agreement between lesion volume estimates. Convolutional neural network (CNN) predictions based on acute phase computed tomography angiography (CTA) and final infarct volumes (**a**), computed tomography perfusion RAPID (CTP-RAPID) ischaemic core (cerebral blood flow, CBF < 30%), and final infarct volumes (**b**) and CTP-RAPID and CNN (**c**)

**Fig. 6:**
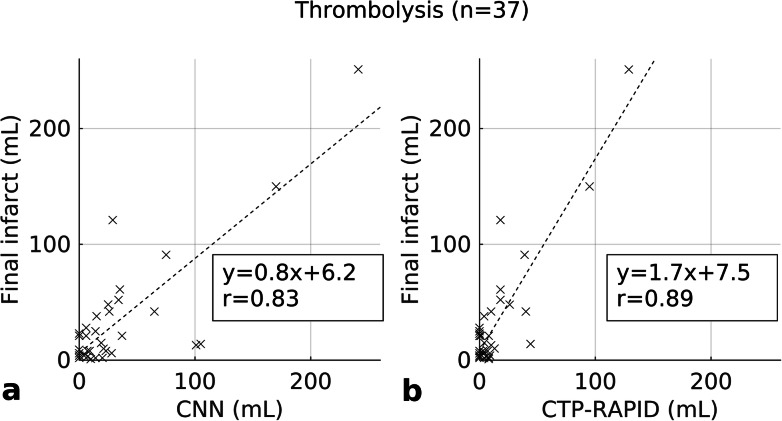
Infarct lesion volume correlation in patients treated with intravenous thrombolysis. Convolutional neural network (CNN) predictions *versus* final infarct volumes (**a**). Computed tomography perfusion RAPID (CTP-RAPID) ischaemic core *versus* final infarct volumes (**b**)

A correlation of *r* = 0.83 (95% CI 0.71–0.91) was found between CNN outputs and manual segmentations of final infarct volumes when all patients were included in the statistical analysis (Fig. 4). The corresponding Bland-Altman plot is shown in Fig. 5 with a mean volume difference of -16.3 (95% limits of agreement -115.0–69.6) mL. A correlation was also found in a subgroup analysis of 57 patients that presented less than 9 h of symptom onset (*r* = 0.79, 95% CI 0.49–0.91), and in a subgroup of 26 patients that presented more than 9 h of symptom onset (*r* = 0.90, 95% CI 0.85–0.96). CTP-RAPID ischaemic core volumes and manual segmentations of final infarct volumes were compared in all patients and a correlation of *r* = 0.91 (95% CI 0.83–0.96) was found. CTP-RAPID tended to underestimate the final infarct volumes (per linear regression, final infarct volume = 1.7 × CTP-RAPID volume +14 mL). The corresponding Bland-Altman plot is shown in Fig. 5 with a mean volume difference of -29.0 (95% limits of agreement -130.4–6.4) mL. The trend in final infarct volume estimation amongst patients who received thrombolytic therapy was closer to ground truth using the CNN (*r* = 0.83, 95% CI 0.25–0.96, slope 0.8) than per CTP-RAPID (*r* = 0.89, 95% CI 0.44–0.97, slope 1.7) as shown in Fig. 6. On average, the CNN-estimated volumes matched more closely to the final infarct volumes in this subgroup (mean volume difference -0.5 mL for CNN and -17 mL for CTP-RAPID), and the overall absolute differences were of the same order (19 and 20 mL, respectively). In this subgroup, 89% of the patients had a predicted volume within 25 mL of the actual follow-up volume with the CNN and 73% with CTP-RAPID. An excellent correlation with final infarct volumes was found in patients who did not receive thrombolytic therapy, with *r* = 0.89 (95% CI 0.80–0.95) and *r* = 0.92 (95% CI 0.83–0.97) for the CNN outputs and the CTP-RAPID estimates, respectively.

**Fig. 7:**
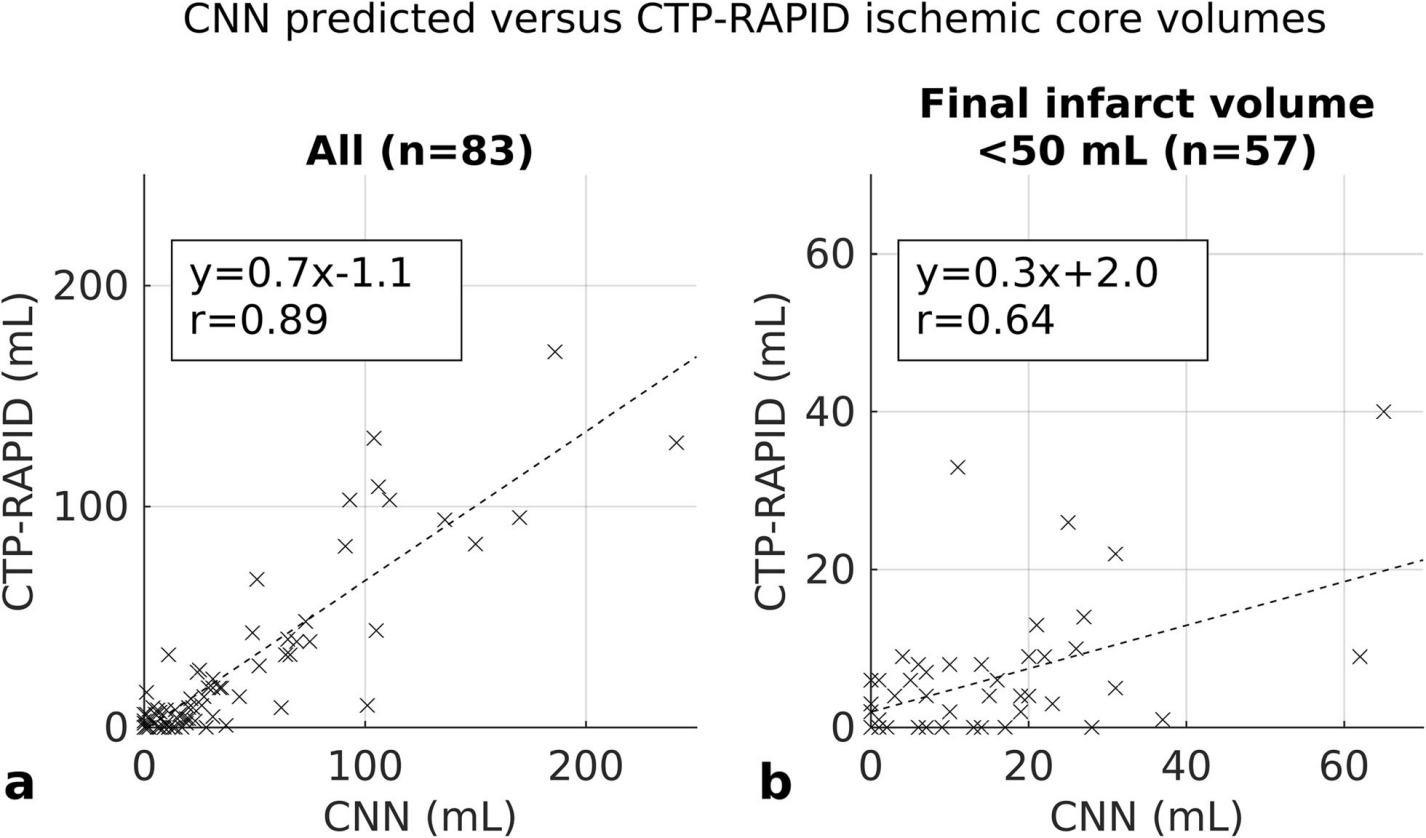
Infarct lesion volume correlation between convolutional neural network (CNN) predictions and computed tomography perfusion RAPID (CTP-RAPID) ischaemic core (cerebral blood flow, CBF < 30%). All cases (**a**). Patients with final infarct volume less than 50 mL (**b**)

To assess the clinical usefulness of our method, the CNN outputs were compared to CTP-RAPID ischaemic core volumes as shown in Fig. 7 and a good correlation was found (*r* = 0.89, 95% CI 0.82-0.94). The corresponding Bland-Altman plot is shown in Fig. 5 with a mean volume difference of -12.7 (95% limits of agreement -81.8–18.5) mL. In a subgroup analysis using the < 9 h time window, a good correlation was found with a tendency of the CNN to overestimate core volumes compared to CTP-RAPID (*r* = 0.90, 95% CI 0.74–0.96; CTP-RAPID volume = 0.6 × CNN volume -1.7 mL). A large proportion of the study population had a final infarct volume of < 50 mL and a correlation of *r* = 0.64 (95% CI 0.30–0.86) was found between the CNN and CTP-RAPID in this group.

Three out of 57 patients in the < 9 h time window were misclassified as not being candidates for thrombolytic therapy according to reported infarct volume, as their CNN reported infarct volumes were greater than 70 mL, with the CTP-RAPID reported ischaemic core being less than 70 mL. No patients were misclassified as having an infarct volume of less than 70 mL when CTP-RAPID classified them in the > 70 mL infarct core group (*n* = 10). This led to an overall sensitivity of 1.00 and specificity of 0.94 for classifying patient eligibility for thrombolytic therapy.

**Fig. 8:**
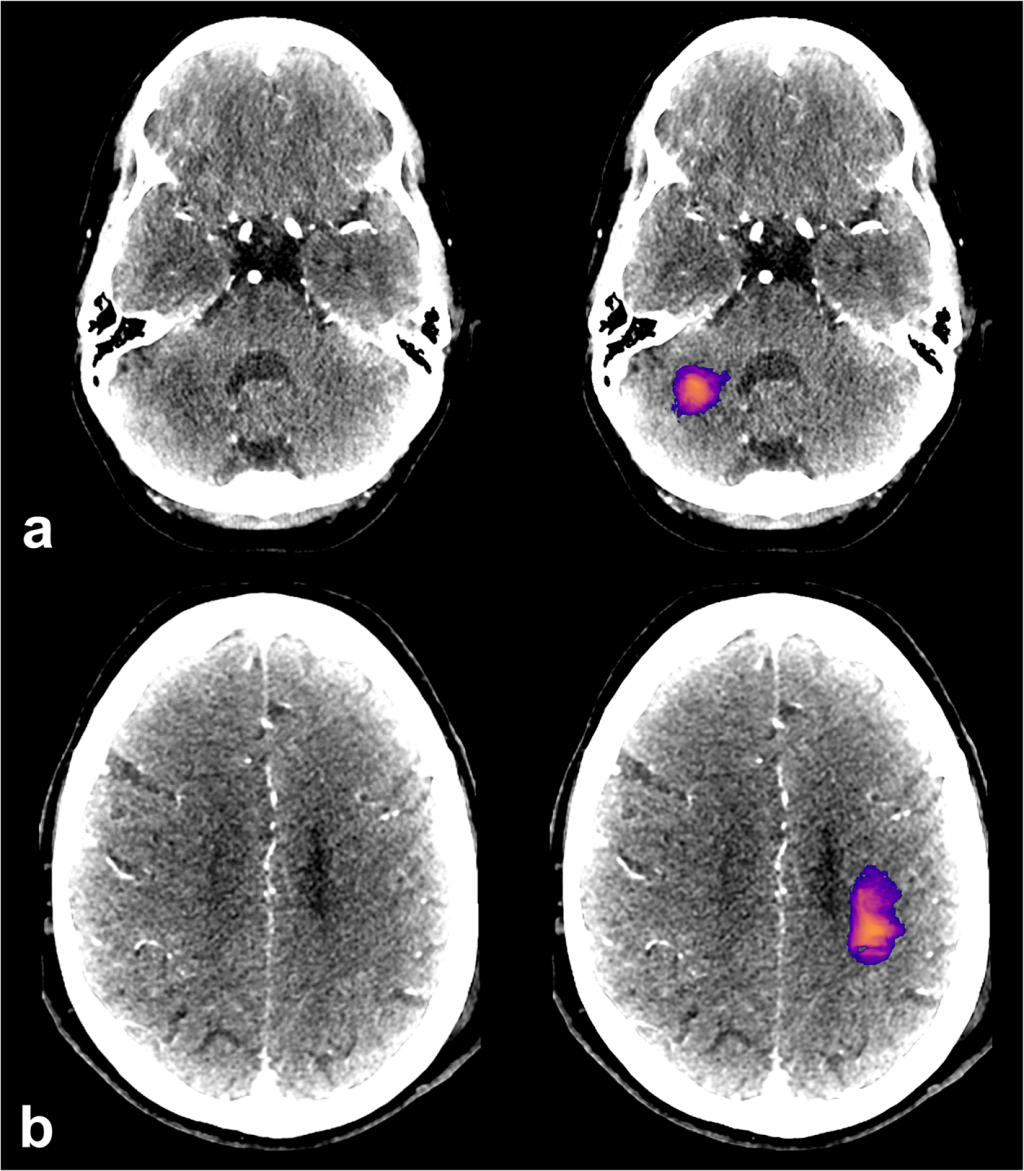
Representative cases of false positive lesions. A lesion caused by beam hardening artefact in the posterior fossa (**a**). A false positive lesion for which no clear radiologic correlation could be found (**b**)

Amongst all 83 patients, a total of 37 (45%) patients had false positive lesions detected by the CNN. False positive lesions in the contralateral cerebral hemisphere were detected in 28 patients, in the posterior fossa in 21 patients and 12 patients had false positive lesions in both areas. There were a total of 71 false positive lesions, and the median size was 1.2 mL (IQR 0.6–2.9 mL, range 0.1–20.9 mL). If only lesions with a volume of > 3mL were included as per CTP-RAPID convention, the number of patients with false positive lesions was reduced to 14 (17%), although this would also result in the exclusion of eight true lesions correctly detected by the CNN. In a visual analysis, we found that 29 false positive lesions were the result of beam hardening artefacts in the frontal, middle and posterior fossa. In 33 cases, no clear abnormality could be detected by the human eye in the areas marked by the CNN. A representation of typical false positive lesions is provided in Fig. 8.

## Discussion

In the present study, we report a good correlation between CNN outputs and manually segmented final infarct volumes from follow-up CT images. The correlation was better in a subgroup that presented more than 9 h after symptom onset, as opposed to a subgroup presenting less than 9 h of symptom onset. This may partly be due to the more even distribution of lesion volumes in the > 9 h subgroup, whereas the < 9 h subgroup had a larger proportion of small lesions. It is also possible that the longer delay from the onset of symptoms to imaging may have led to irreversible ischaemic brain tissue hypoattenuation that is not as strongly affected by circulatory conditions, e.g., iodine concentration in CTA-SI as may be in the earlier time window.

ischaemic lesions delineated in CTA-SI have been shown to correlate with infarct core as shown in diffusion-weighted imaging [14, 15], which is regarded as the gold standard. However, these studies have been conducted with previous generation scanners and a number of more recent studies have postulated that modern CT-scanners with rapid image acquisition, faster contrast-injection rates of 5–7 mL/s and short prep-delay times of 15–20 s may lead to CTA being more CBF than cerebral blood volume weighted, resulting in overestimation of the infarct core [16–19]. This has been attributed to the brain tissue not reaching a steady-state in contrast opacification before image acquisition. Our study is in line with these findings in that we found a tendency for the CNN to overestimate infarct volumes compared to the CTP-RAPID core volumes as shown in Fig. 7, whilst the overall correlation was good. On the other hand, our CNN tended to underestimate infarct sizes when compared to final infarct volumes in patients who did not receive thrombolytic therapy. This may owe to infarct growth in situations where recanalisation does not happen and also to ischaemia-related oedema, which reaches its peak at one to several days after the onset of ischaemia as tissue water content increases [20–22]. In keeping with previous studies, a tendency to overestimate final infarct volumes was found in a subgroup of patients treated with thrombolytic therapy, suggesting that CTA also reflects penumbral tissue to an extent.

The CNN seemed not to be able to detect ischaemic lesions in the caudate nucleus and in the basal ganglia area generally (Fig. 3), which may be due to the small initial training set. Also, the CNN usually did not detect ischaemic lesions in the most peripheral zone of the cerebral hemispheres, including the cortical grey matter. This is usually an area of the brain most opacified by contrast material and would be expected to express ischaemic hypoattenuation better than white matter, where attenuation differences between normal and ischaemic brain may be as low as 3–4 Hounsfield units and obscured by image noise [23]. This phenomenon might be explained by the location near hyperdense calvaria and partly by ischaemia-related oedema changing the anatomical relationships and thus hindering the performance of rigid registration between the CTA and the follow-up CT images. Somewhat surprisingly, the CNN seemed to be relatively insensitive to chronic white matter hypointensities or chronic parenchymal defects, as only 9 false positive lesions were attributed to chronic white matter hypointensities due to vascular degeneration or to chronic infarcts.

Although a stronger correlation was found amongst all patients between CTP-RAPID and final infarct volumes than with CNN outputs and final infarct volumes, the CNN still had good correlation with final infarct volumes (*r* = 0.83) and also with CTP-RAPID core volumes (*r* = 0.89). A previous study reported that their CNN prediction probabilities from CTA images corresponded with CTP-RAPID ischaemic core volumes with *r* = 0.7 [8]. As opposed to our study, they trained their algorithm against dichotomised CTP-RAPID determinations of ischaemic core without comparing the CNN outputs to follow-up imaging, only to CTP-RAPID at presentation. Their study design included a patient population balanced to contain comparable numbers of patients with small, moderate and large-sized ischaemic cores at presentation whereas we selected consecutive patients resulting in a large number of smaller ischaemic cores, which is a confounding factor in the statistical analysis of our results. The results of these studies suggest that a CTA-based CNN method could be helpful in treatment selection using the clinical-imaging mismatch approach [24].

We tested the clinical relevance of our method by comparing the CNN outputs to CTP-RAPID core volumes and found a good correlation between the two with a tendency of the CNN to overestimate core volumes compared to CTP-RAPID. Three out of 57 patients in the < 9-h time window with CTP-RAPID volume < 70 mL were misclassified as not being candidates for thrombolytic therapy, so the overall specificity was 0.94 for classifying patient eligibility for thrombolytic therapy. However, the trend in final infarct volume estimation amongst patients who received thrombolytic therapy was closer to ground truth per the CNN than per CTP-RAPID. This may be due to the fundamental differences in these two methods, i.e., CTP-RAPID estimating volumes based on circulation and complex algorithms, whereas CTA estimates of infarct core are based on brain tissue hypoattenuation. Because ischaemic hypoattenuation on CTA has been found to include some penumbra as discussed above, the uncertain and possibly incomplete and slower recanalisation (as compared to endovascular thrombectomy) achieved with i.v. thrombolysis may be reflected in the results. However, the evolution of an infarct is known to be a complex matter and there are numerous variables affecting the final outcome, which are beyond the scope of this text [22]. CTP-based core estimation methods also have their own inherent weaknesses and it has been questioned whether CTP should be used to make treatment decisions in individual patients [25]. Furthermore, in our series, six out of the initial patient cohort of 91 had a technically unsuccessful CTP but a successful CTA.

Although the mean volume difference between CNN outputs and final infarct volumes was smaller than between CTP-RAPID and final infarct volumes, the 95% limits of agreement were broad with both methods. Previously, the CTP-based method of treatment selection has been questioned in individual patients for its high variability in core volume estimation between subjects [25]. In larger study populations, however, the average performance justifies its use [3]. A similarly high variability was also found in our study. Based on the Bland-Altman plots in Fig. 5, the performance of our CNN method is similar to the CTP-based RAPID in this regard. This should be taken into account in possible clinical use, as there is a possibility of under or overestimating the core volume, which may in turn affect treatment selection.

Limitations of the present study include its single-centre retrospective design and a limited sample size, which is especially apparent in the subgroup analyses. Also, the majority of the CTA studies were performed using the same scanner, which limits the generalisability of the results across different centres and scanners. The small size of the initial CNN training data set (*n* = 30) can also be considered a limitation. Finally, a follow-up CT was used for segmentation of final infarct volumes. A follow-up study using diffusion-weighted imaging would have been more accurate, but CT is routinely used for follow-up in our institution for several reasons, including costs and patient tolerance.

In conclusion, our study showed that a CTA-based CNN model can detect anterior circulation acute ischaemic lesions and provide good estimates for infarct core volumes.

## Data Availability

The datasets used and/or analysed during the current study are available from the corresponding author on reasonable request.

## Abbreviations

AIS: Acute ischaemic stroke
ASPECTS: Alberta stroke programme early computed tomography score
CI: Confidence interval
CNN: Convolutional neural network
CT: Computed tomography
CTA: CT angiography
CTA-SI: CTA source images
IQR: Interquartile range
SD: Standard deviation
